# QuEChERS-超高效液相色谱-串联质谱法测定全血中65种合成大麻素

**DOI:** 10.3724/SP.J.1123.2025.04037

**Published:** 2026-04-08

**Authors:** Zhang XI, Yi YE, Songpan LI, Jing KANG, Weiqun LIU, Jing ZHANG, Daoxia LI

**Affiliations:** 1.成都民用航空医学中心，四川 成都 610016; 1. Chengdu Civil Aviation Medical Center，Chengdu 610016，China; 2.四川大学华西基础医学与法医学院，四川 成都 610065; 2. West China College of Basic Medicine and Forensic Medicine，Sichuan University，Chengdu 610065，China; 3.四川省食品检验研究院，四川 成都 610097; 3. Sichuan Provincial Institute of Food Inspection and Research，Chengdu 610097，China; 4.四川大学华西第四医院，四川 成都 610065; 4. West China Fourth Hospital of Sichuan University，Chengdu 610065，China

**Keywords:** 合成大麻素类, 超高效液相色谱-串联质谱, QuEChERS法, 蛋白沉淀法, 动态多反应监测, synthetic cannabinoids, ultra performance liquid chromatography-mass spectrometry （UPLC-MS/MS）, QuEChERS, protein precipitation, dynamic multiple reaction monitoring （dMRM）

## Abstract

合成大麻素已成为世界上新精神活性物质中涵盖物质种类最多、滥用最严重的一类物质，为了对合成大麻素滥用情况进行检测，本研究建立了同时测定全血中65种合成大麻素的超高效液相色谱-串联质谱（UPLC-MS/MS）方法。研究对前处理及检测条件进行了优化，采用基质匹配标准曲线内标定量方式，实现了全血中65种合成大麻素的快速筛查和定量分析。全血采用乙腈沉淀蛋白后，经QuEChERS萃取净化，以0.1%甲酸水溶液-0.1%甲酸乙腈溶液为流动相，经Waters Acquity UPLC HSS T_3_柱（100 mm×2.1 mm，1.8 μm）分离，流速0.25 mL/min，柱温40 ℃，进样量2 μL，动态多反应监测。结果表明，全血中65种合成大麻素在0.05~200 ng/mL范围内线性关系良好（相关系数（*r*）>0.992），检出限为0.01~0.2 ng/mL，定量限为0.05~0.5 ng/mL，满足实际样品分析需求。以不含合成大麻素的空白血为基质，在1、5、50 ng/mL 3个加标水平下进行加标回收试验，各目标物回收率为62.2%~116.9%，日内、日间精密度均小于10%，基质效应为70.2%~117.7%，稳定性较好，具有稀释可靠性。使用本研究建立的检测方法对10份大麻吸食人群血液样品进行筛查，10份样品中均检出了目标化合物，检出化合物分别为ADB-BUTINACA、MDMB-4en-PINACA、MDMB-FUBICA和5F-MDMB-PICA，含量范围为1.9~23.1 ng/mL，其中ADB-BUTINACA和MDMB-4en-PINACA检出率较高，分别为90%和50%。此外，在6份血样中同时检出2种或3种合成大麻素。研究结果表明所建方法具有准确、快速、灵敏及分离效果较好等优点，可应用于毒品检验检测机构对全血中合成大麻素的快速筛查和定量分析，可为打击毒品犯罪、维护社会稳定提供强有力的技术保障。

## Abstract (en)

Synthetic cannabinoids （SCs） have become the most diverse and widely abused class of new psychoactive substances in the world. Blood and urine are classic sample matrices for in vivo toxin analysis， but some SCs have high lipophilicity， and the concentration of parent drugs in urine is extremely low， making them difficult to detect. Metabolites need to be investigated， and SCs with similar structures can produce the same metabolites. Therefore， establishing urine detection methods and interpreting results are relatively complex. In contrast， the detection of synthetic cannabinoid parent drugs in blood is more straightforward， and detecting parent drugs in blood can serve as direct evidence in legal cases. In order to detect the abuse of SCs， a QuEChERS-ultra performance liquid chromatography-tandem mass spectrometry （UPLC-MS/MS） method was developed to simultaneously determine 65 SCs in blood. The sample preparation and detection conditions were optimized. Quantification was achieved using an internal standard and matrix-matched calibration curves， enabling rapid screening and quantitative analysis of the 65 SCs in blood. Following protein precipitation with acetonitrile， the blood extract was further extracted and purified using QuEChERS reagents. Chromatographic separation of all 65 SCs was performed on a Waters Acquity UPLC HSS T_3_ column （100 mm×2.1 mm， 1.8 μm） maintained at 40 ℃. Detection employed dynamic multiple reaction monitoring （dMRM） mode with a mobile phase consisting of 0.1% （volume fraciotn） formic acid aqueous solution and acetonitrile， delivered at a flow rate of 0.25 mL/min. The injection volume was 2 μL. The 65 SCs exhibited good linear relationships in the mass concentration range of 0.05-200 ng/mL with correlation coefficents （*r*） exceeding 0.992. The limits of detection （LODs） were between 0.01 and 0.2 ng/mL， and the limits of quantitation （LOQs） were between 0.05 and 0.5 ng/mL， respectively， which meet the requirements for analyzing SCs in blood sample. The intra-day and inter-day precisions （*n*=6） were determined to be 1.0%–9.9% by spiking blank blood samples with the 65 SCs at mass concentrations of 1， 5， and 50 ng/mL. The recoveries of the 65 SCs were between 62.2% and 116.9%， and the matrix effects were between 70.2% and 117.7%. All analytes demonstrated good stability and acceptable dilution integrity in blood samples. Using the established method， 10 blood samples from suspected drug use cases were successfully screened for SCs. The target compounds were detected in all 10 samples， specifically including ADB-BUTINACA， MDMB-4en-PINACA， MDMB-FUBICA， and 5F-MDMB-PICA with mass concentrations ranging from 1.9 to 23.1 ng/mL. The detection rates of ADB-BUTINACA and MDMB-4en-PINACA were 90% and 50%， respectively， suggesting their relatively high prevalence in China’s illegal drug market. In addition， two or more SCs were detected simultaneously in six blood samples. Compared with other existing literature methods， this study combines QuEChERS with precipitation protein method for blood sample pretreatment， greatly improving the detection efficiency and suitable for rapid screening of whole blood samples in batches. The results demonstrate that the established method offers accuracy， rapidity， sensitivity， and effective chromatographic separation. It can serve as a reliable tool for forensic laboratories to perform rapid screening and quantitative analysis of SCs in blood， providing robust technical support for combating drug-related crime and supporting social stability.

合成大麻素类（SCs）是人工合成的大麻素受体（CBR）激动剂，可与体内大麻素受体1（CB1R）或大麻素受体2（CB2R）结合，产生比天然大麻素更强的生理药理效果^［1，2］ ^。SCs较天然大麻对中枢神经系统、心血管系统、免疫系统的副作用更大，过量吸食会导致中毒甚至死亡^［3］ ^。由于SCs相较于种植大麻更容易获取，目前已成为全世界新精神活性物质（new psychoactive substances，NPS）中涵盖物质种类最多、滥用最严重的一类物质^［4］ ^。据报道，SCs最早于2004年在欧洲开始作为传统毒品的替代品在互联网上销售^［5，6］ ^，2008年首次在“香料”中检测到^［7］ ^，之后SCs陆续蔓延到其他国家^［8］ ^。SCs通常以散装粉末、片剂形式制造和运输，使用有机溶剂溶解后喷洒在烟丝、花瓣等植物表面，在市场上以“K_2_”“香料”、干花、小树枝、巧克力、饼干等具有伪装性的形式进行出售^［5，6］ ^。近年来，在电子烟油中检测到SCs的报道较多，这种包装形式的时尚性对青少年有极大的吸引力^［9-11］ ^，使青少年成为SCs滥用的高危人群。目前全世界范围内已报告了多起滥用SCs导致的大规模中毒和死亡案件^［12-14］ ^，严重影响人类的身心健康和公共卫生安全。另外，关于吸食SCs后发生的暴力伤害致死及性侵等刑事案件时有报道^［15-18］ ^，对公共秩序和社会治安构成重大威胁。

从体内检材中得出的毒物分析结果可用作法医学鉴定的直接依据，血液和尿液是经典的样本基质，但部分SCs的亲脂性较强，原型物质在尿液中的浓度极低，难以检测，需要进行代谢物的考察，而结构类似的合成大麻素会产生相同的代谢产物，因此尿液检测方法的建立和结果解释较为复杂^［19-22］ ^。相比之下，针对血液中合成大麻素母体药物的检测更加便捷，并且血液中原型物质的检出可作为司法案件中的直接证据^［23］ ^。

本研究通过超高效液相色谱-串联质谱（UPLC-MS/MS）技术，综合近年来收集到的合成大麻素滥用案件中定性检出阳性的化合物以及国内外文献报道的新增合成大麻素等^［24-27］ ^，选择了包括吲哚衍生物、吲唑衍生物、吡咯衍生物、吡唑衍生物等在内的65种合成大麻素作为目标物，建立了全血中合成大麻素的检测方法并进行方法学考察，并通过对大麻吸食人群的血液样品进行检测以考察方法的实用性。本研究旨在建立有效的合成大麻素检测手段，为司法案件中涉毒人员的查证、毒品的管制与鉴定、毒品犯罪的认定与惩治提供数据参考和理论支持。

## 1 实验部分

### 1.1 仪器与试剂

Agilent LC1290-6460液相色谱-三重四极杆质谱仪（配有电喷雾离子源，美国Agilent公司）；N1-50氮吹仪（中国杭州奥盛科学仪器有限公司）；MS 3旋涡振动器（德国IKA公司）；H1650-W台式高速离心机（湖南湘仪仪器有限公司）；Oasis HLB固相萃取小柱（美国Waters公司）；0.22 μm有机滤膜（北京泰科瑞迪国际贸易有限公司）。

65种合成大麻素标准品及内标物2-甲基-1-丙基-3-（1-萘甲酰基）吲哚-d_7_（JWH-015-d_7_）均购自上海原思标物科技有限公司，质量浓度均为1 mg/mL。甲醇、乙腈、甲酸（色谱纯，德国Merck公司）；无水硫酸钠、无水硫酸镁、碳酸钠、碳酸氢钠（分析纯，成都市科隆化学品有限公司）；Milli-Q超纯水纯化系统（美国Millipore公司）。

### 1.2 标准溶液配制

分别取65种合成大麻素标准品适量，用乙腈配制质量浓度为1.0 μg/mL的混合标准储备液，于-20 ℃避光保存。使用前以乙腈为稀释剂，将混合标准储备液逐级稀释为1 000、500、250、50.0、25.0、5.0、2.5、0.50和0.25 ng/mL的混合标准工作液。

精密吸取JWH-015-d_7_标准溶液20 μL，使用乙腈定容至20 mL，配制质量浓度为1 μg/mL内标标准储备液，于-20 ℃避光保存。使用前以乙腈为稀释剂，配制质量浓度为50 ng/mL的内标工作液。

### 1.3 仪器条件

#### 1.3.1 色谱条件

Acquity UPLC HSS T_3_色谱柱（100 mm×2.1 mm，1.8 μm）；流动相A为0.1%甲酸水溶液，流动相B为0.1%甲酸乙腈；梯度洗脱程序：0~16.0 min，30%B~90%B；16.0~18.0 min，90%B；18.0~18.2 min，90%B~30%B；18.2~20.0 min，30%B；流速0.25 mL/min；柱温 40 ℃；进样量2 μL。

#### 1.3.2 质谱条件

具体质谱条件、65种合成大麻素及内标的质谱参数见文献［^27^］。

### 1.4 样品溶液制备

取200 μL全血样品于15 mL离心管中，加入内标工作液0.1 mL，涡旋混匀20 s。加入0.9 mL乙腈后，再加入400 mg无水MgSO_4_，振荡10 min，以10 000 r/min离心10 min，取上清液过0.22 µm有机滤膜，待UPLC-MS/MS进样分析。

### 1.5 基质匹配标准溶液制备

精密吸取空白全血200 μL，除不添加标准品和内标溶液外以相同方法对空白全血进行前处理获得空白基质。分别精密吸取混合标准工作溶液适量，并加入内标工作液0.1 mL，用空白基质定容至1 mL，制备成折算到全血样品中的质量浓度范围为0.05~200 ng/mL的系列基质匹配标准溶液。

### 1.6 加标质控样品溶液制备

精密吸取空白全血200 μL，添加适量混合标准工作溶液，配制成低、中、高3个质量浓度（1、5、50 ng/mL）的全血添加质控样品（内标质量浓度为5 ng/mL），补足乙腈总量为1 mL，其余与样品溶液同法制备即得加标质控样品溶液。

## 2 结果与分析

### 2.1 质谱条件优化

动态多反应监测（dMRM）是一种特殊的多反应监测方式，以目标化合物的保留时间为中心设置采集时间窗，质谱仅在设定的时间窗口内采集相应的MRM数据^［28，29］ ^。根据设定色谱条件下各目标物的保留时间等信息建立65种SCs基于dMRM的质谱方法，这种科学的采集方式通过对保留时间窗口变化范围进行自动分配，可有效提高整个采集时间内的分析效率和灵敏度。dMRM模式下65种SCs及内标物的提取离子流图见附图1（www.chrom-China.com）。

### 2.2 色谱条件优化

选择合适的色谱柱和流动相对多化合物的检测具有重要意义，比较了色谱柱Waters Acquity UPLC HSS T_3_ （100 mm×2.1 mm，1.8 μm）、ACE Excel 2 Super C_18 _（75 mm×2.1 mm，2 μm）和Agilent Eclipse Plus C_18_（50 mm×3.0 mm，1.8 μm）的分离效果。结果表明，使用ACE C_18_和Agilent C_18_色谱柱时，AM-2233、5F-BEPIRAPIM、AM-1220等物质出峰较早，保留时间在3 min以内，受柱压及流动相比例变化影响较大，峰形较差；Agilent C_18_色谱柱不能通过保留时间实现对同分异构体5F-7-APAICA和5F-APINACA的区分。对于极性较小的目标化合物，3种色谱柱均可获得对称性好的色谱峰，无明显拖尾，但ACE和Agilent色谱柱对出峰时间早的物质检测效果不佳。相较而言，Waters Acquity UPLC HSS T_3_色谱柱作为分析柱时可延长AM-2233、5F-BEPIRAPIM、5F-APINACA等化合物的保留时间，减少目标化合物重叠，有利于今后在此基础上增加新的目标化合物，并能有效分离5F-7-APAICA（保留时间11.92 min）和5F-APINACA（保留时间15.29 min），3种色谱柱对10 ng/mL全血添加样品中合成大麻素的分离效果见附图1。

由于不同骨架结构的合成大麻素极性存在较大差异，且部分合成大麻素具有氨基等碱性官能团，会与色谱柱内二氧化硅颗粒表面的硅醇基团相互作用^［12］ ^，因此选择0.1%甲酸水溶液和0.1%甲酸乙腈作为流动相，在流动相中加入甲酸作为质子供体有助于抑制硅醇基团电离，乙腈作为有机相具有较强的洗脱能力和较低的背景噪声。在进行多化合物检测时，优先选取梯度洗脱的方式进行色谱分离，通过提高水相初始比例至70%，可延长AM-2233、5F-BEPIRAPIM、AM-1220等物质的保留时间，避免目标物质出峰过早，受到溶剂效应的影响。通过调整流动相梯度，实现了ADB-BINACA、MMB-FUBICA等化合物与结构相似目标物的有效分离，同时内标与目标物质之间不会产生干扰，此外异构体5F-7-APAICA和5F-APINACA可以根据保留时间的差异进行区分。

### 2.3 样品前处理方法的选择

目前已报道的全血中SCs测定的前处理方法主要有沉淀蛋白法（protein precipitation， PPT）^［10］ ^、QuEChERS法^［11］ ^、液-液萃取法（liquid-liquid extraction， LLE）^［30］ ^和固相萃取法（solid-phase extraction， SPE）^［31］ ^。本研究对4类前处理方法的提取回收率进行了考察。

考察方法：分别取空白血200 μL，加入混合标准储备液适量制备成50 ng/mL的全血质控样品，分别按下列方法进行前处理，每种方法平行制备3份。

PPT法：于全血质控样品中，加入内标工作液0.1 mL，涡旋混匀20 s，补加乙腈至1 mL，振荡10 min，以10 000 r/min离心10 min；取上清液，过0.22 µm有机滤膜，待进样分析。

QuEChERS法：于全血质控样品中，加入内标工作液0.1 mL，涡旋混匀20 s，补加乙腈至1 mL，再加入400 mg无水MgSO_4_，振荡10 min，以10 000 r/min离心10 min，取上清液过0.22 µm有机滤膜，待进样分析。

LLE法：于全血质控样品中，加入内标工作液0.1 mL，涡旋混匀20 s，加入1 mL碳酸氢钠缓冲液（pH 10.2），混匀后加入5 mL正己烷-乙酸乙酯（98∶2，体积比），提取两次，合并上清液在40 ℃氮气流下蒸发至干燥，用1 mL的50%乙腈水溶液（含0.1%甲酸）复溶，过0.22 µm有机滤膜，待进样分析。

SPE法：于全血质控样品中，加入内标工作液0.1 mL，涡旋混匀20 s，加入2 mL蒸馏水，旋涡混匀2 min，以10 000 r/min离心10 min。参考Rosado等^［31］ ^的研究，分别用6 mL乙酸乙酯、3 mL甲醇和3 mL超纯水对Oasis HLB固相小柱（60 mg/3 mL）进行预处理。将离心后的样品装入固相小柱，用2 mL甲醇-水（5∶95，体积比）进行淋洗。抽真空15 min后用6 mL乙酸乙酯洗脱样品。洗脱液在40 ℃氮气流下吹干，并用1 mL的50%乙腈水溶液（含0.1%甲酸）复溶，过0.22 µm有机滤膜，待进样分析。

结果表明，LLE法的回收率最低，为5.29%~58.55%；SPE法回收率为5.33%~125.97%。采用SPE虽然能对目标化合物进行纯化和富集，但洗脱液的选择和溶剂的比例需进一步优化，固相萃取法处理后大部分物质的提取回收率在80%以上，但5F-ABICA、AB-BICA、5F-ADBICA、ADB-BICA、ADB-FUBICA和CB-13等物质的回收率低于50%，表明研究选择的试剂和条件难以兼顾所有物质；PPT法提取回收率为56.83%~134.68%；QuEChERS法的提取回收率为64.73%~123.43%，提取回收率更高且更稳定，结果见图1。另外，LLE法和SPE法均需氮吹并复溶，操作步骤比沉淀蛋白法和QuEChERS法更复杂，样品处理耗时较长，因此本研究采取QuEChERS法进行前处理。

**图1:**
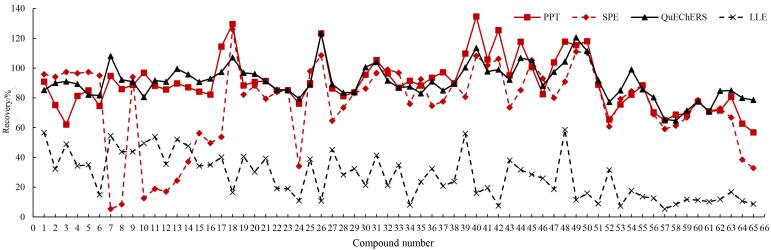
不同前处理方法处理后全血中65种合成大麻素的加标回收率比较（*n*=3）

### 2.4 QuEChERS法前处理条件优化

QuEChERS法中常加入硫酸盐作为脱水剂和盐析剂，可以吸收全血中的水分，并增强水相的离子强度，提高SCs在提取溶剂的分配系数，进而改善回收率。而全血中含有大量蛋白质，会影响SCs的提取效率，因此本研究在文献［^11^］基础上结合蛋白沉淀法，对前处理条件进行优化，优化内容包括蛋白沉淀剂和硫酸盐种类的选择。

#### 2.4.1 蛋白沉淀剂的选择

UPLC-MS/MS仪器要求沉淀剂具有高挥发性，以避免残留干扰离子化过程。乙腈和甲醇因其挥发性强、沉淀效率高而成为首选，可通过离心快速去除蛋白，同时减少色谱柱污染。研究以回收率为评价指标，考察了甲醇、乙腈和甲醇乙腈混合溶液（1∶1，体积比）沉淀蛋白时对65种目标物提取效率的影响，结果如图2和图3所示，除部分含有吲哚-3-甲酰胺结构的化合物，如ADB-FUBICA、AB-BICA、ADB-FUBINACA、PX-2等20种化合物外，其余化合物在3种沉淀剂作用下回收率无显著差异，而DB-FUBINACA、PX-2等化合物在乙腈条件下回收率显著高于另外两种溶剂，可能是由于甲酰胺基团极性较强并具有一定的碱性，与其他类别合成大麻素的溶解性存在较大差异造成的，综合考虑65种化合物的提取回收率，选择乙腈作为提取溶剂兼蛋白沉淀剂。

**图2:**
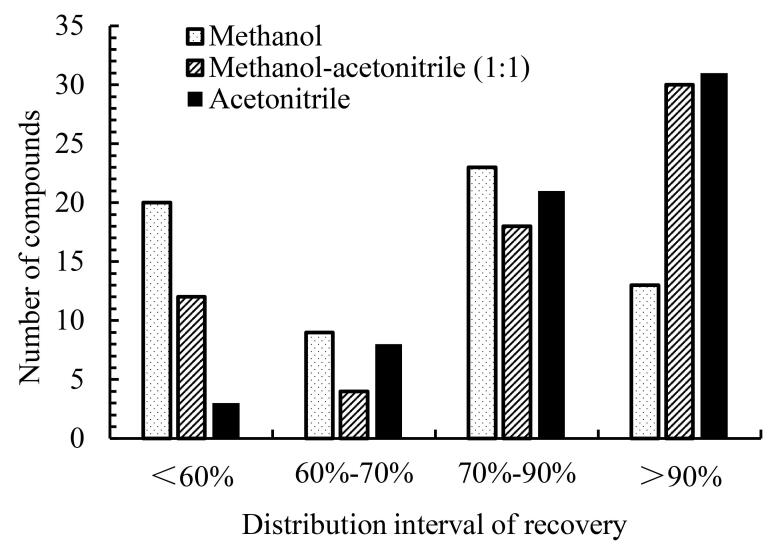
不同蛋白沉淀剂处理后全血中65种合成大麻素回收率分布

**图3:**
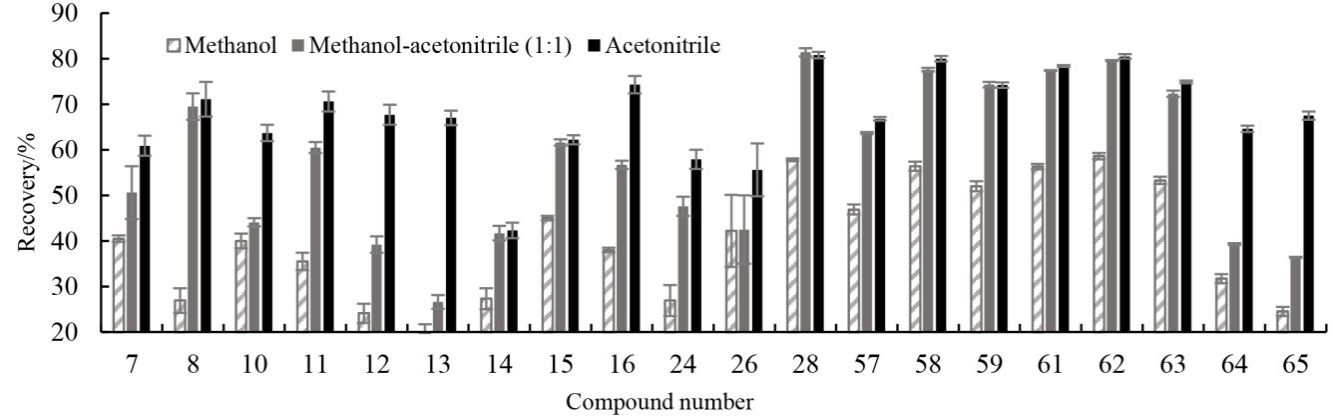
不同蛋白沉淀剂处理后全血中20种合成大麻素的加标回收率（*n*=3）

#### 2.4.2 硫酸盐种类选择

QuEChERS法中常用的硫酸盐有无水Na_2_SO_4_和无水MgSO_4_，研究考察了0.4 g无水Na_2_SO_4_和无水MgSO_4_的效果。结果如图4所示，两种脱水剂对绝大部分目标物影响基本一致，但使用无水MgSO_4_的回收率相对较高，其中回收率大于60%的化合物占比为95.4%（62/65），无水Na_2_SO_4_组回收率大于60%的化合物占比为87.7%（57/65）；同时无水MgSO_4_条件下3，5-AB-CHFUPYCA等16种目标物的回收率显著优于无水Na_2_SO_4_，如图5所示。可能是无水Na_2_SO_4_脱水过程中出现结块现象，导致目标物被包裹，进而影响提取回收率，综合65种化合物的提取效率，选用MgSO_4_作为脱水剂和盐析剂。

**图4:**
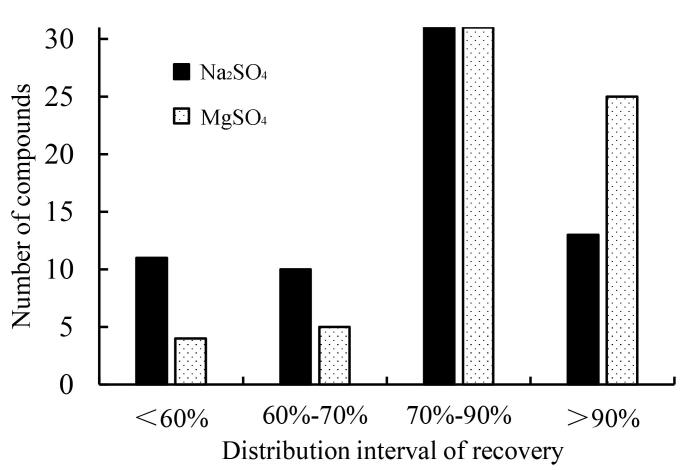
不同硫酸盐处理后全血中65种合成大麻素回收率分布

**图5:**
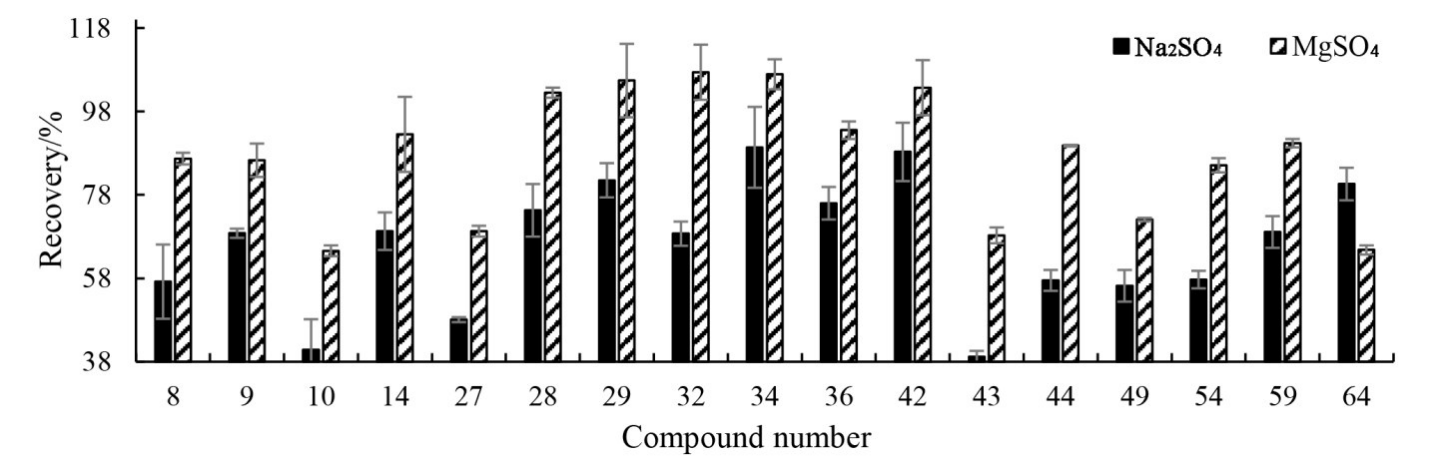
不同硫酸盐处理后血中16种合成大麻素的加标回收率（*n*=3）

### 2.5 方法学验证

#### 2.5.1 选择性考察

取6个不同来源的空白全血样品各200 μL，不添加待测物和内标，进行前处理后直接进样获得空白全血的色谱图，考察内源性物质对待测物和内标的干扰。将一定浓度的混合标准溶液和内标溶液加入空白全血中，进行前处理后进样分析。结果表明，血中内源性物质在目标物和内标的出峰时间段内无干扰信号，方法具有较好的选择性，能够满足分析要求。

#### 2.5.2 标准曲线和定量范围

将质量浓度为0.05、0.1、0.5、1、5、10、50、100和200 ng/mL的基质标准溶液，按照低浓度到高浓度的顺序进样。以样品质量浓度（*X*）为横坐标，定量离子的峰面积与内标峰面积之比（*Y*）为纵坐标拟合标准曲线。不断稀释混合标准溶液添加到全血样品中，前处理后进样，以信噪比为3为标准，确定各物质的检出限（LOD），以信噪比为10确定出定量限（LOQ）。全血样品中65种合成大麻素的检出限和定量限见表1。结果表明，65种合成大麻素的LOD为0.01~0.2 ng/mL，LOQ为0.05~0.5 ng/mL。各SCs的线性范围均为0.1~200 ng/mL，加权系数为1/*X*，相关系数为0.992~1.000，线性关系良好。

**表1:** 全血中合成大麻素的保留时间、检出限、定量限、基质效应和回收率（*n*=6）

No.	SCs （CAS No.）	*t*_R_/min	LOD/（ng/mL）	LOQ/（ng/mL）	1 ng/mL		5 ng/mL		50 ng/mL	
					ME/%	RE/%	ME/%	RE/%	ME/%	RE/%
1	AM-2233 （444912-75-8）	3.20	0.01	0.05	90.0	84.8	85.2	89.8	84.3	83.9
2	5F-BEPIRAPIM （2365471-74-3）	3.54	0.01	0.05	85.0	100.6	98.0	96.0	100.0	95.0
3	AM-1220 （137642-54-7）	3.81	0.05	0.1	84.9	89.0	89.1	92.5	98.9	86.1
4	JWH-200 （103610-04-4）	3.87	0.01	0.05	90.0	106.7	98.0	95.1	82.5	95.8
5	A-796260 （895155-26-7）	4.35	0.05	0.1	85.5	101.3	95.8	100.0	94	96.1
6	AM-1248 （335160-66-2）	5.39	0.05	0.1	88.9	79.2	77.8	70.9	74.9	71.8
7	5F-ABICA （1801338-26-0）	5.97	0.2	0.5	83.2	111.9	99.7	110.6	106	94.6
8	AB-BICA （1969264-37-6）	6.37	0.2	0.5	87.6	93.4	93.4	94.2	103.7	89.8
9	5-fluoropentyl-3-pyridinoylindole （HCl） （2365471-19-6）	6.58	0.05	0.2	103.2	93.0	90.3	97.8	115.7	93.9
10	5F-ADBICA（1801338-27-1）	6.98	0.05	0.1	117.7	100.9	91.0	106.6	106.4	100.8
11	ADB-BICA （2219319-40-9）	7.37	0.2	0.5	109.5	102.9	88.2	96.4	98.0	92.1
12	ADB-FUBICA （1801338-23-7）	7.56	0.2	0.5	114.3	99.7	89.5	99.7	114.2	86.3
13	PX-2 （2365471-47-0）	7.56	0.2	0.5	108.8	105.4	85.2	106.3	113.3	99.2
14	5-CI-AB-PINACA （1801552-02-2）	7.65	0.2	0.5	111.0	104.0	89.5	108.2	115.7	107.7
15	ADB-BINACA （1185282-27-2）	7.98	0.05	0.2	113.1	100.7	80.0	102.5	91.9	98.6
16	ADB-FUBINACA （1185887-21-1）	8.12	0.2	0.5	104.5	89.3	87.8	100.0	105.8	99.8
17	ADB-BUTINACA （2682867-55-4）	8.18	0.05	0.1	92.6	105.7	92.8	114.2	106.1	96.0
18	5F-SDB-005 （2185863-14-1）	8.29	0.05	0.1	82.6	109.5	103.9	110.4	109.9	107.6
19	ADBICA （1445583-48-1）	8.78	0.1	0.2	103.7	89.8	86.0	103.5	98.0	100.1
20	5F-7-QUPAIC （2748300-92-5）	9.31	0.01	0.1	71.9	113.4	76.8	116.3	75.6	109.8
21	ADB-PINACA （1633766-73-0）	9.57	0.2	0.5	114.2	100.2	91.9	106.8	98.6	101.9
22	MEP-FUBICA （2749985-70-2）	10.04	0.05	0.1	93.7	110.2	82.3	108.4	89.1	104.9
23	MMB-FUBICA （1971007-90-5）	10.31	0.05	0.1	94.1	103.4	83.7	109.1	90.1	105.4
24	5F-MPP-PICA（2001-05-21）	10.25	0.01	0.1	107.3	105.3	90.3	109.1	99.8	108.1
25	MMB-022 （2659308-31-1）	10.40	0.05	0.2	99.7	116.7	98.4	108.8	99.0	107.5
26	SDB-005 （1801552-01-1）	10.55	0.1	0.2	110.0	103.5	99.0	91.1	95.5	102.9
27	5F-MDMB-PICA （1971007-88-1）	10.64	0.01	0.05	98.0	95.7	93.0	85.7	100.0	100.6
28	3，5-AB-CHMFUPPYCA （1870799-79-3）	10.78	0.1	0.3	94.8	91.5	85.7	93.9	89.5	95.3
29	MAB-CHMINACA （1863065-92-2）	10.80	0.2	0.5	104.8	99.5	89.6	107.2	97.4	97.0
30	5F-NNEI （1445580-60-8）	11.01	0.01	0.05	77.2	105.4	72.3	102.2	77.7	98.8
31	5F-AMB （1801552-03-3）	11.01	0.01	0.05	83.9	110.1	84.6	111.5	78.9	106.7
32	MDMB-FUBICA （1971007-91-6）	11.17	0.01	0.05	79.4	113.4	77.5	115.8	77.9	109.0
33	5F-CUMYL-PICA （1400742-18-8）	11.19	0.01	0.05	73.3	103.3	77.2	110.2	80.0	107.0
34	MMB-FUBINACA （1971007-92-7）	11.21	0.05	0.2	80.6	112.2	78.8	116.9	82.0	111.0
35	SDB-006 （695213-59-3）	11.22	0.01	0.1	77.3	110.3	78.2	113.3	71.2	111.7
36	5F-MDMB-PINACA （1715016-75-3）	11.90	0.01	0.1	97.9	108.3	89.3	108.4	94.0	105.4
37	5F-7-APAICA （2682867-58-7）	11.92	0.05	0.1	101.5	102.2	86.2	99.0	94.4	97.7
38	FUBIMINA （1984789-90-3）	12.49	0.01	0.05	77.6	92.7	79.2	97.2	78.7	91.1
39	MMB-CHMICA （1971007-94-9）	12.60	0.01	0.05	97.3	98.6	88.9	100.0	101.1	99.3
40	RCS-4 （1345966-78-0）	12.84	0.01	0.05	84.9	105.4	82.5	106.0	86.0	102.6
41	JWH-015 （155471-08-2）	12.86	0.01	0.05	76.5	102.4	77.4	97.7	79.4	96.1
42	FUB-JWH-018 （2365471-45-8）	12.95	0.1	0.3	82.5	97.3	81.5	93.8	82.6	95.7
43	MDMB-4en-PINACA （2504100-70-1）	13.02	0.01	0.05	87.0	98.2.0	87.0	103.6	86.7	95.4
44	5F-MN18 （1445581-91-8）	13.19	0.05	0.15	81.8	112.9	103.1	110.8	115.8	114.4
45	JWH-250 （864445-43-2）	13.36	0.01	0.05	71.2	99.5	79.0	101.6	75.9	97.7
46	CUMYL-PEGACLONE （2160555-55-3）	13.66	0.01	0.05	71.5	99.7	77.2	102.5	79.2	97.8
47	MDMB-CHMICA （1971007-95-0）	13.82	0.05	0.1	84.8	95.2	86.7	101.4	94.4	98.3
48	NM-2201 （2042201-16-9）	14.14	0.1	0.3	70.4	108.7	78.0	108.0	71.9	104.5
49	JWH-203 （864445-54-5）	14.2	0.05	0.1	80.0	102.7	76.5	99.6	74.9	96.6
50	FDU-PB-22 （1883284-94-3）	14.49	0.2	0.5	77.2	89.2	71.5	95.1	77.9	92.2
51	JWH-018 （209414-07-3）	14.64	0.01	0.05	76.9	85.6	89.6	78.2	81.6	81.3
52	JWH-018 BENZIMIDAZOLE ANALOG （2316839-70-8）	14.86	0.01	0.05	70.2	89.1	77.6	91.2	77.2	90.5
53	JWH-007 （155471-10-6）	15.13	0.01	0.05	77.7	85.6	76.4	83.8	78.3	86.0
54	5F-APINACA （1400742-13-3）	15.29	0.01	0.05	87.9	91.9	89.9	87.5	90.5	85.4
55	JWH-307 （914458-26-7）	15.37	0.01	0.05	92.5	85.5	92.8	84.9	93.5	83.8
56	MDMB-CHMINACA （1185888-32-7）	15.45	0.01	0.05	109.1	88.8	105.0	94.7	102.4	89.2
57	APICA （1345973-50-3）	15.63	0.01	0.05	94.5	80.6	98.2	85.8	93.2	79.6
58	RCS-8 （1345970-42-4）	15.71	0.01	0.05	93.8	86.2	99.6	93.7	90.2	81.2
59	FUB-APINACA （2180933-90-6）	15.73	0.05	0.1	100.8	82.1	102.1	99.2	95.3	82.4
60	5F-APINAC （2365471-88-9）	16.18	0.05	0.1	115.3	92.2	108.0	96.5	103.0	86.4
61	JWH-210 （824959-81-1）	16.33	0.01	0.05	92.5	87.4	94.9	89.1	88.9	76.5
62	JWH-370 （914458-22-3）	16.37	0.05	0.1	91.6	82.1	94.2	90.2	88.4	79.6
63	5CI-APINACA （2160555-52-0）	16.41	0.05	0.2	98.4	79.1	112.1	86.3	110	81.5
64	EG-018 （2219320-91-7）	17.55	0.05	0.1	90.3	69.3	94.3	68.2	83.7	69.6
65	CB-13 （432047-72-8）	18.33	0.2	0.5	85.0	62.2	89.9	67.0	81.5	69.3
/	JWH-015-d_7_ （/）	12.78	/	/	/	/	/	/	/	/

#### 2.5 3　回收率

向空白血样中加入合成大麻素标准溶液，制备低、中、高3个质量浓度（1、5、50 ng/mL）的全血质控样品，对全血添加样品的回收率（RE）进行考察，每个浓度制备6个平行，计算回收率。结果如表1所示，各物质的回收率为62.2%~116.9%，表明该方法具有较好的准确性和可靠性。

#### 2.5 4　精密度考察

制备低、中、高3个质量浓度（1、5、50 ng/mL）的全血质控样品，每种浓度的质控样品在一日内早、中、晚各取3份进行测定，每份全血样品重复测定3次考察日内精密度，并在连续3天的相同时间点进样考察日间精密度。精密度以测定浓度的相对标准偏差（RSD）表示。日内精密度和日间精密度结果见附表1。实验结果表明各物质的日内精密度均小于9.0%，日间精密度均小于9.9%，所有物质的精密度均小于15%，表明该方法具有良好的精密度。

#### 2.5 5　基质效应的评价

取空白全血进行前处理获得空白基质。参考文献［^32^］，选择低、中、高3个质量浓度（1、5、50 ng/mL）评估目标化合物的基质效应。结果如表1所示，65种SCs的基质效应为70.2%~117.7%，其中78.5%（51/65）的目标化合物基质效应为80%~120%，而JWH-018、JWH-250、JWH-203、5F-AMB、SDB-006等14种合成大麻素在3种浓度下的基质效应均小于80%，具有弱离子抑制效应。内标法虽然能够在一定程度上弥补基质效应，但本方法采用的内标物质非对应化合物的同位素内标，由于化合物性质差异等因素，方法所选内标物未能完全弥补上述14种目标物的基质效应；为了定量结果更加准确、可靠，本方法同时采用内标物和基质匹配标准曲线进行定量。

#### 2.5 6　稳定性考察

参考ICH《M10：生物分析方法验证及样品分析》^［33］ ^对全血添加样品的稳定性进行考察，制备1 ng/mL和50 ng/mL加标空白全血添加样品（内标质量浓度为5 ng/mL），进行以下条件的稳定性考察：室温放置24 h；-20 ℃条件下3次冻融循环（每次循环间隔为24 h）；-20 ℃、-80 ℃条件下放置30天。各考察条件下平行制备3份样品，以各时间点测定值与放置时间为0 h时样品测定值之间的偏差（Bias）为指标进行考察。

结果（见附表2）表明，室温放置24 h及经过3次冻融循环后待测物质的偏差均在±20%范围内，精密度小于10%，表明分析物在此两种条件下能够保持稳定。在-20 ℃、-80 ℃条件下放置30天后大多数物质的偏差在±20%范围内，但5F-BEPIRAPIM、AM-1220、ADB-BINACA、5F-SDB-005、5F-MPP-PICA、5F-AMB、MMB-CHMICA、5F-MN18、FDU-PB-22、JWH-007、JWH-307、JWH-210等物质的损失超过20%，损失较大，提示采样后应尽快进行检测，不宜放置过长时间。

#### 2.5 7　稀释可靠性考察

由于部分目标物存在弱基质效应，同时部分研究曾报道有少数样品呈现非常高的浓度^［12-14］ ^，超出本研究所建曲线的最高质量浓度（200 ng/mL），因此有必要对稀释可靠性进行考察。

用空白基质对质量浓度为500 ng/mL的质控样品进行5倍、10倍和20倍稀释，将测得浓度与实际浓度进行比较，结果见附表3。结果表明，稀释5倍、10倍和20倍后测得的浓度精密度值均小于14.0%，大多数物质的测得量与理论浓度之间的偏差在±15%以内，符合ICH《M10：生物分析方法验证及样品分析》中对生物样本稀释可靠性的要求，表明稀释后测得的浓度是准确可靠的，实际检测过程中遇到高浓度样本时，可采用空白基质稀释后进行测定。

### 2.6 方法对比

将本方法与现有文献报道的血液中SCs的分析方法进行了对比，见表2。

**表2:** 本方法与其他方法的比较

Matrix	Number of analytes	Pre-treatment methods	Analytical method	LOD/（ng/mL）	LOQ/（ng/mL）	Ref.
Blood	65	PPT/QuEChERS	LC/MS/MS	0.01-0.2	0.05-0.5	this study
Blood	15	QuEChERS	LC/MS/MS	0.4-16	/	［^9^］
Blood	132	PPT	LC/MS/MS	/	0.25	［^34^］
Serum	30	LLE	LC/MS/MS	0.01-2.0	0.1-2.0	［^35^］
Blood	3	SPE	LC/MS/MS	0.04-0.14	0.10-0.21	［^36^］
Blood	51	PPT/online SPE	LC-LIT-MS	0.02-1	0.1-4	［^37^］
Blood	10	QuEChERS	UPLC-QE-Orbitrap-MS	0.05-0.05	0.05-0.2	［^38^］
Blood	23	PPT	DART-MS/MS	0.01-1	10	［^39^］

LIT： linear ion trap； QE： quadrupole/electrostatic； DART： direct analysis in real time.

在方法的前处理方式上，单纯采用沉淀蛋白的处理方式通常无法去除全血中的盐；液液萃取的提取效率与缓冲液的选择、溶液碱性强弱程度有关，固相萃取的洗脱液和溶剂比例往往难以兼顾所有合成大麻素的测定。另外，液-液萃取法和固相萃取法均需氮吹并复溶，操作步骤比沉淀蛋白法和QuEChERS法更复杂，样品处理的耗时较长。相比而言，QuEChERS法具有快速、高效的特点，使用无水MgSO_4_作为脱水用盐可增强水相的离子强度，降低合成大麻素的水溶性，提高其在乙腈内的溶解度和提取回收率，该方法与沉淀蛋白法相结合用于全血样品前处理，极大地提高了检测效率，适合全血样品的批量快速筛查。

### 2.7 实际样品测定

利用本方法对收集到的大麻吸食人群的血液样品进行检测，检出情况如表3所示。

**表3:** 血液样品中合成大麻素的检出情况

Sample No.	Contents of SCs/（ng/mL）			
	MDMB-4en-PINACA	ADB-BUTINACA	5F-MDMB-PICA	MDMB-FUBICA
1	6.0	10.1		
2	13.9	9.2		
3				3.1
4		8.3		
5		6.0	16.9	
6		9.2		
7		7.1		
8	5.1	17.8		
9	23.1	1.9	2.2	
10	18.6	20.1		

由表3可看出，共检出4种目标物，其中ADB-BUTINACA（90%，9/10）和MDMB-4en-PINACA（50%，5/10）检出率较高，表明这两种物质在我国非法药物市场上较为流行，且6份样品中同时检测出2种或3种物质，提示合成大麻素有混合使用的可能，该结果与前期研究中毛发样品检测结果^［27］ ^的趋势基本一致。

## 3 总结

本文利用UPLC-MS/MS技术，基于动态多反应监测模式，建立了全血中65种合成大麻素的检测方法，目标化合物基本涵盖了近年来报道的可能滥用的合成大麻素，可在20 min内对65种目标物进行定性、定量分析，同分异构体5F-7-APAICA和5F-APINACA实现了有效分离。经实际样品验证，本方法具有准确、快速、灵敏等优点，适用于全血样品中65种合成大麻素的快速筛查和定量分析。该方法可推广到相关毒品检验检测单位，为打击毒品犯罪、维护社会稳定提供强有力的技术保障。
